# Laser-assisted vessel welding: state of the art and future outlook

**Published:** 2015-09-30

**Authors:** Dara R. Pabittei, Wadim de Boon, Michal Heger, Rowan F. van Golen, Ron Balm, Dink A. Legemate, Bas A. de Mol

**Affiliations:** 1 Department of Cardiothoracic Surgery, Academic Medical Center, University of Amsterdam, Amsterdam, the Netherlands; 2 Department of Surgery, Academic Medical Center, University of Amsterdam, Amsterdam, the Netherlands; 3 Department of Physiology, Faculty of Medicine, Hasanuddin University, Makassar, South Sulawesi, Indonesia; 4 Department of Experimental Surgery, Academic Medical Center, University of Amsterdam, Amsterdam, the Netherlands; 5 Department of Biomedical Engineering, Material Technology, Technical University Eindhoven, Eindhoven, the Netherlands

**Keywords:** laser-assisted vessel welding, suture anastomosis, laser-assisted vascular anastomosis, thermodynamic, tissue, heat distribution

## Abstract

Laser-assisted vascular welding (LAVW) is an experimental technique being developed as an alternative to suture anastomosis. In comparison to mechanical anastomosis, LAVW is less traumatic, non-immunogenic, provides immediate water tight sealant, and possibly a faster and easier procedure for minimally invasive surgery. This review focuses on technical advances to improve welding strength and to reduce thermal damage in LAVW. In terms of welding strength, LAVW has evolved from the photothermally-induced microvascular anastomosis, requiring stay sutures to support welding strength, to sutureless anastomoses of medium-sized vessels, withstanding physiological and supraphysiological pressure. Further improvements in anastomotic strength could be achieved by the use of chromophore-containing albumin solder and the employment of (biocompatible) polymeric scaffolds. The anastomotic strength and the stability of welds achieved with such a modality, referred to as scaffold- and solder-enhanced LAVW (ssLAVW), are dependent on the intermolecular bonding of solder molecules (cohesive bonding) and the bonding between solder and tissue collagen (adhesive bonding). Presently, the challenges of ssLAVW include (1) obtaining an optimal balance between cohesive and adhesive bonding and (2) minimizing thermal damage. The modulation of thermodynamics during welding, the application of semi-solid solder, and the use of a scaffold that supports both cohesive and adhesive strength are essential to improve welding strength and to limit thermal damage.

## 1. Introduction

Laser-induced vessel bonding is an attractive alternative for suture anastomosis. The technique is based on the sutureless coaptation of vessel segments by photothermal [1-47] or photochemical [48] processes. Photothermal laser vessel bonding currently comprises the most frequently employed technique and can be divided into two distinct modalities, namely native laser-assisted vascular welding (LAVW) [1-45] and modalities where proteinaceous solders are used to adjoin vessel segments, namely solder-enhanced LAVW (sLAVW) [49-67].

Compared to conventional suture anastomosis (CSA), the potential advantages of laser vascular bonding include a reduction in foreign body reaction, liquid-tight sealing (i.e., immediate closure of the incision or wound that prevents bleeding or seeping of fluids from the perfused blood vessels), faster healing, and simpler and more rapid alternatives for minimally invasive and endoscopic anastomotic techniques [1-3,5,7,8, 10-12,14,16,17,22-24,27,28,30,32,35,36,40,44,45,68,69].

During photothermal LAVW the radiant energy is converted to heat by the tissue’s endogenous chromophores (e.g., water or pigments) or by exogenously applied chromophores in the solder, causing denaturation of proteins and consequent bonding of tissue. The ultimate goal of LAVW is to obtain a coaptation that can withstand supraphysiological pressure (> 250 mmHg) with minimum thermal damage to the vessel wall.

**Figure 1.**
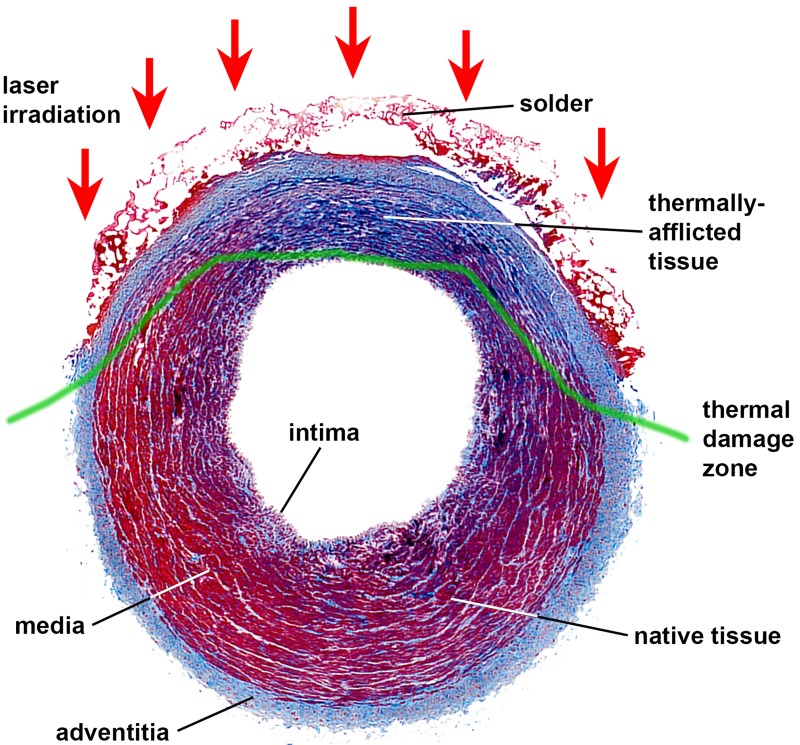
Photomicrograph of an ssLAVW-treated porcine carotid artery, showing the vascular layers, area of laser irradiation, thermally afflicted tissue, thermal damage zone and non-affected native tissue. A Masson’s trichrome stain was used to determine laser-induced structural alterations in connective tissue (collagen stains light blue, damaged collagen stains dark blue-to-purple, and solder, elastin, and muscle fibers stain red). Note that the ssLAVW resulted in marked shrinkage of the vascular wall and differential staining (i.e., damage) in the thermally afflicted tissue.

**Figure 2.**
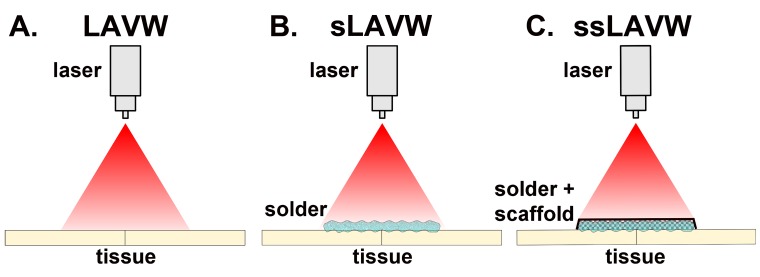
Schematic drawing of (A) laser-assisted vascular welding (LAVW), (B) solder-based LAVW (sLAVW), and (C) scaffold- and solder-enhanced LAVW (ssLAVW).

Unfortunately, several drawbacks associated with current (native) LAVW techniques [6,18,20,26,34,44,45] have hampered its transition from the experimental to the clinical setting [40,44,45]. First, the relatively low welding strengths produced with LAVW often require additional sutures to reinforce the anastomosis, ultimately defeating the purpose of the modality. [3,5-8,10,12-14,18,20,22,24,26,28,30,32,34-36,40,67,69,70] Second, extensive thermal damage may extend to the basal membrane of the intima (Figure 1) and cause intimal hyperplasia [7, 20], thrombosis [7, 26], and aneurysm formation [13, 15,21,34,38,71,72]. To overcome these disadvantages, several more refined welding techniques have been developed, including sLAVW, scaffold- and solder-enhanced LAVW (ssLAVW) (Figure 2), and photochemical laser-assisted tissue bonding.

This review therefore summarizes the techniques recently introduced to improve welding strength and reduce thermal damage in microvessels to medium-sized vessel anastomoses. Furthermore, the current status of laser-induced vascular bonding, the pros and cons of each LAVW modality, and technical improvements that are required to further advance LAVW are addressed. Based on this information, a recommendation is made regarding an optimal welding modality for future in vivo and clinical use.

## 2. Data collection

A literature search was performed in the Medline database. The following keywords were used in singular form or as Boolean operators: laser-assisted vascular anastomosis, LAVA, laser-assisted vessel repair, LAVR, laser welding, solder, vessel, scaffolds, electrospinning (electrical charge-based technique to produce nonwoven scaffold from a liquid polymer), solvent casting and particulate leaching (a technique to produce membrane scaffolds by dissolving a polymer in organic solvent), and biodegradable polymers. Relevant articles in which welding had been performed on the urethra, intestine, colon, skin, and nerves were included in the initial screening. Manual cross-referencing was performed where necessary to retrieve additional data.

## 3. Photothermal laser-assisted vessel welding modalities

### 3.1. Laser-assisted vessel welding

#### 3.1.1. Principal mechanism

Vascular anastomosis or repair by LAVW is based on the photothermal denaturation of collagens [1-3,5-7,9-12,14-23, 26-31,33-38,69-75]. The welding process begins with the delivery of light to closely adjoined vessels (Figure 2A) and subsequent absorption and conversion of photons to heat by an endogenous chromophore (i.e., water and pigments). The increase in local temperature induces structural alterations in vessel wall proteins, which leads to protein cross-linking and consequent coaptation of the vessel segments. Although the exact types of structural alterations that result in welding are currently elusive [40,44,67], collagen cross-linking is considered to play a major role [4,33,40,44]. The extent to which these interprotein bonds are formed dictates the welding strength [4,33,40,44].

LAVW has mainly been employed to either make vascular end-to-end, end-to-side, or side-to-side anastomoses or to repair damaged vasculature (i.e., to join two aortic strips or to close a longitudinal vessel wall incision). Throughout this review the term LAVW refers to both types of welding, whereas the terms laser-assisted vascular anastomosis (LAVA) and laser-assisted vascular repair (LAVR) refer to the specific regimens.

#### 3.1.2. Laser-tissue interactions

Laser welding is a rate-dependent process that comprises (1) heat generation, (2) heat diffusion, and (3) temperature-dependent alteration of the molecular structure of tissue constituents. The increase in temperature and the distribution of heat in the vessel wall determines the amount of collagen cross-linking and thus the welding strength. This whole process is linearly proportional to time and exponentially proportional to temperature [56,76].

**Figure 3.**
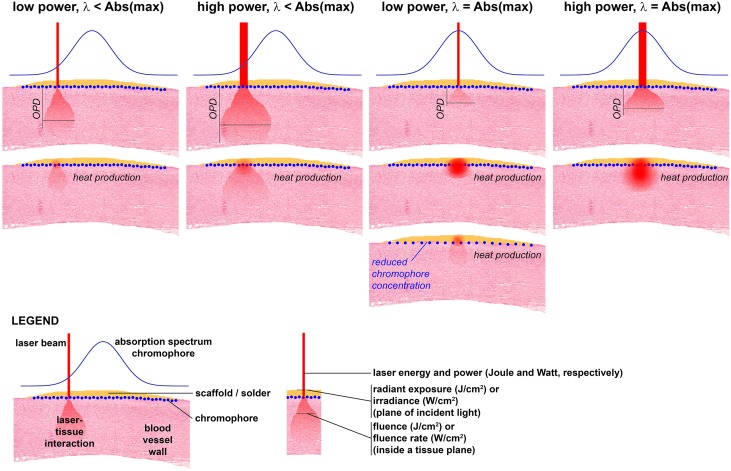
General fundamentals of laser-tissue interactions. Four main scenarios are presented, each depicted in a column. The top row provides a schematic illustration of optical penetration depth (OPD) for every scenario, whereas the bottom row reflects the corresponding heat production in the solder/scaffold and tissue. The scenarios are the following: (1) low-power laser beam of a wavelength (λ) that is lower than the absorption maximum (Abs(max)) of the chromophore (i.e., moderate heat production at chromophore layer, moderate optical penetration depth, moderate thermal spread in tissue); (2) high-power laser beam of a λ that is lower than the Abs(max) of the chromophore (i.e., moderate-to-high heat production at chromophore layer, large optical penetration depth, high thermal spread in tissue); (3) low-power laser beam of a λ that is equal to the Abs(max) of the chromophore (i.e., extensive heat production at chromophore layer, low optical penetration depth, thermal spread mainly confined to scaffold/solder- tissue interface); (4) high-power laser beam of a λ that is equal to the Abs(max) of the chromophore (i.e., very profound heat production at chromophore layer, low-to-moderate optical penetration depth, extensive thermal spread at scaffold/solder- tissue interface with heat diffusion laterally and into tissue). In the third row of the third column, a scenario is presented in which the chromophore concentration is reduced, accounting for moderate heat production at chromophore layer, moderate optical penetration depth, moderate thermal spread in tissue. The legend in the bottom left corner explains every component in each panel. Moreover, the common nomenclature is provided at the bottom center of the figure.

Lasing parameters (i.e., wavelength (λ), irradiance (W/cm^2^), irradiation time (s), and irradiation mode (continuous-wave or pulsed laser)) along with water (chromophore) and macromolecular components of the tissue govern the generation of heat in the vessel wall (Figure 3). The deposition of radiant energy in the tissue is dependent on tissue’s scattering index (SI) and absorption coefficient. The SI is controlled by the macromolecular structure of the tissue, whereas the absorption coefficient is dictated by laser wavelength and the water content in the tissue. Water acts as an endogenous chromophore that absorbs and converts incident light to heat [40,44,76]. Typically, laser penetration in water (i.e., optical penetration depth (OPD)) increases with decreasing wavelength. For example, the infrared CO_2_ laser wavelength (λ = 10,600 nm) is predominantly absorbed by water, resulting in a superficial OPD, whereas the visible and near-infrared wavelengths of diode lasers (**λ** = 670-988 nm) exhibit a significantly greater OPD in tissue. The absorption of some lasers can be selectively enhanced by pigment molecules (i.e., hemoglobin and melanin) [20,21,30,36, 72,77] or exogenous dyes, such as indocyanine green (ICG) [78] and fluorescein isothiocyanate [53]. The degree of heat build-up is determined by the laser irradiance (W/cm^2^, i.e., power output (W) / spot area (cm^2^)), radiant exposure (J/cm^2^, irradiance × irradiation time (s)), and the lasing mode (i.e., continuous or pulsed mode). Furthermore, the temporal changes in thermal profile within the tissue are dictated by the molecular composition of the tissue.

The thermodynamic variations during welding fuel the constant discussion regarding which exact types of bonding are responsible for LAVW [44]. The different laser wavelengths, lasing parameters, and tissue molecular compositions produce different thermodynamic profiles. Heath-induced collagen denaturation starts at a temperature of ~60 °C and becomes irreversible when tissue reaches a temperature of ~64 °C [79]. Thus, if lasing is terminated at around 70-75 °C, welding is achieved by collagen cross-linking. Apart from collagen denaturation, thermal damage also starts at approximately 60 °C (i.e., via cell necrosis) and peaks at temperatures of > 80 °C, as characterized by the increased in membrane permeability [40,76]. Thus, the main objectives of LAVW are to induce sufficient collagen cross-linking and to minimize thermal damage. To obtain these goals, welding temperature should be kept in the range of 65-75 °C. Accordingly, the proper means to modulate thermodynamics to obtain the desired temperature include: (1) using a laser wavelength with an OPD that is similar to the thickness of the tissue, (2) efficient energy absorption in the tissue with minimum scattering, and (3) a sufficient energy output for collagen denaturation [33]. The fundamental principles underlying laser-tissue interactions in the context of (ss)LAVW are summarized in Figure 3.

#### 3.1.3. Summary of experimental results

**Figure 4.**
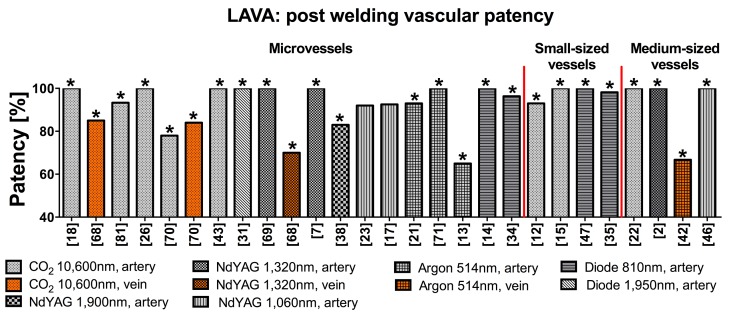
Summary of the percentage of immediate patency obtained with LAVA using different laser wavelengths on vessels of various sizes. All welds were performed with the use of stay sutures unless indicated (*). Microvessels, small-, and medium-sized vessels indicate vessel sizes of ≤ 1 mm (i.e., vessels of a rat), 1-2 mm (i.e., vessels of a rabbit), and 2-7 mm (i.e., vessels of a pig), respectively.

The main advantages of LAVA over CSA include the elimination of mechanical trauma and suture materials, which reduces inflammatory responses, foreign body reactions, and thrombosis. However, in most LAVA studies, stay sutures were still employed to support vessel approximation and anastomotic strength (Figure 4) [2,7,12-15,21-23,27,31,34,35,38,42, 43,46,47,68-71,81]. Several studies in which stay sutures were entirely omitted and the vessel segments were adjoined using microforceps, a balloon catheter, or a polyvinyl alcohol splint reported lower patency rates and were not applicable to all vessel sizes [16,17,23].

**Figure 5.**
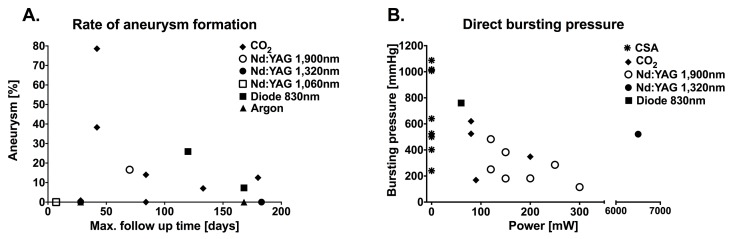
(A) Aneurysm rate plotted as a function of follow-up time after LAVA at different laser wavelengths and (B) immediate welding strengths (expressed as bursting pressure) as a function of laser power.

The outcomes of LAVA (i.e., patency, anastomotic strength, thermal damage, and complications) are primarily governed by lasing parameters and the type and thickness of the target tissue. Figure 4 demonstrates the superior patency of arterial anastomoses compared to venous anastomoses. The superficial absorption of the CO_2_ laser (λ = 10,600 nm) was more suitable for microvascular anastomoses (Ø ≤ 1 mm). However, when operated in continuous-wave mode, CO_2_ LAVW was often associated with full-thickness thermal damage and a high rate of aneurysm formation (Figure 5A) [1,7,12,26,43,70,72, 80,81]. LAVW with a pulsed thulium-holmium-chromiumdoped (THC):yttrium-aluminum-garnet (YAG) laser (λ = 2,150 nm, targets water as chromophore), which has an OPD between that of the CO_2_ and neodymium (Nd):YAG laser (λ = 1,064 nm, also targets water as chromophore), produced sutureless microvascular anastomoses with bursting pressures of 400 ± 55 mmHg, while thermal damage was limited to the adventitia [39].

Unlike continuous-wave lasing, pulsed lasing provides better control over heat deposition and prevents excessive heat build-up, thereby reducing the extent of thermal damage [39]. The Nd:YAG laser was the preferred laser for both microvessels and small-sized vessels (Ø = 0.6-1.2 mm). Sutureless LAVA with an Nd:YAG laser resulted in good patency and partial thermal damage. Diode lasers (λ = 810-988 nm, target water as chromophore) produced supraphysiological welding strength (Figure 5B) but exhibited full-thickness thermal damage in microvessel LAVA. The thermal damage was associated with an increased rate of thrombosis and aneurysm formation [14,31,34,35]. Diode lasers are therefore more suitable for LAVA of medium-sized vessels. To prevent excessive heat generation, LAVW with argon lasers (hemoglobin absorption) is often performed in conjunction with saline irrigation during welding. LAVW with saline irrigation resulted in lower welding temperatures (i.e., 50 °C vs. 80 °C for CO_2_ LAVW) [21,30, 36,71,77] and was associated with decreased risk of thrombosis [30].

Furthermore, welding strength is dictated by power output, irradiance, and radiant exposure [38,78]. A lower power output is favorable because this typically produces higher welding strengths (Figure 5B) [5,7,18,26,35,38]. Irradiance and pulse duration are the most important parameter to define thermodynamics during welding. Unfortunately, not many studies report these parameters.

#### 3.1.4. Drawbacks of LAVW

Despite numerous efforts to accommodate lasing parameters to the type and thickness of tissue, LAVW is still far from clinical application. In most instances, stay sutures are required to obtain a welding strength that is comparable to suture anastomoses. In fact, in the only clinical study on vessel welding, LAVW was employed as an adjunct to suture anastomosis, [82] which largely nullifies the entire purpose of LAVW. In that respect, Bass et al. [40] posited that, unless welding strength can be improved while eliminating the use of stay sutures, LAVW cannot be used for the purposes of making primary anastomoses [40]. Furthermore, ambiguous endpoints, inconsistent results, and extensive thermal damage remain major drawbacks that hinder the transition to the clinical setting [40,56].

### 3.2. Solder-based laser-assisted vessel welding (sLAVW)

#### 3.2.1. Principal mechanism

To eliminate the requirement of stay sutures and to improve welding strength, protein solders have been employed to facilitate laser tissue welding. Procedurally, a thin layer of protein solder, either in liquid, semi-solid, or solid state is applied on the interface of the adjoined vessel segments. Laser irradiation denatures both solder and tissue proteins, resulting in cross-linking of proteins in the solder and the adventitia and consequent adhesion of the coagulated solder to the vascular wall. Hence, the fortifying effect of sLAVW is derived from the bonding between solder and tissue proteins (adhesive bonding) and the bonding between solder proteins (cohesive bonding) [40].

Solders may be photocoagulated with a water-targeting laser (e.g., CO_2_ laser [67]) or with visible lasers using a wavelength-specific chromophore dissolved in the solder [24,49-51, 53-55,57,58,60,61,63-66,83,84]. The chromophore enables the absorption of visible light, causing heat generation in exclusively the solder layer, thereby shielding the deeper vascular layer from thermal damage when proper laser parameters are employed. Indocyanine green (ICG) and methylene blue (MB) are the most commonly used chromophores in sLAVW. Other chromophores that have been used in sLAVW include fluorescein isothiocyanate [53] and food colorings (i.e., red #40, blue #1, and green consisting of yellow #5 and blue #1) [85].

#### 3.2.2. Laser-solder interactions

As with LAVW, the thermodynamics in sLAVW involve the absorption of laser light by the chromophore-containing solder, heat generation, and heat diffusion throughout the solder and into the vascular layer. The possibility to predetermine the absorption coefficient (by the choice of chromophore) and to modulate chromophore concentration has made chromophoreattuned visible light lasers the normative choice for sLAVW.

**Figure 6.**
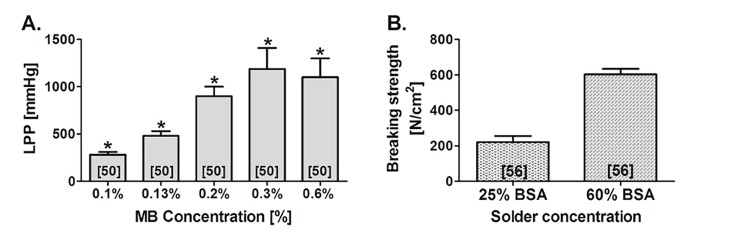
Welding strength obtained by sLAVR performed with (A) different concentrations of methylene blue (MB) and (B) different concentrations of bovine serum albumin (BSA). The welding strength is expressed as leaking point pressure (LPP) or breaking strength. In (A) MB was dissolved in 41% porcine serum albumin (PSA) solder and sLAVR was performed with a 670-nm diode laser. In (B) sLAVR was performed with an 808-nm diode laser and indocyanine green as chromophore (0.25 mg/mL BSA solder). (*) indicates that stay sutures were used during sLAVR. The numbers in brackets indicate the reference from which the data were obtained.

The goal of sLAVW is to predominantly deposit heat at the solder-tissue interface in order to ensure protein denaturation and subsequent cross-linking between solder proteins and adventitial proteins. Several factors play an important role in sLAVW. First, light should preferably penetrate through the entire solder in order to ensure sufficient heat generation at the solder-tissue interface. Consequently, the chromophore density in the solder is critical for achieving optimal welding strength. The optimal chromophore concentration is defined as the concentration at which absorption in the solder is roughly homogeneous across the full thickness of the solder. An intrasolder chromophore density that is too low will result in subcritical heat production and insufficient protein denaturation, yielding low welding strength. In contrast, an intrasolder chromophore density that is too high will induce optical shielding and extreme heating in the upper solder layer, resulting in charring and/or over-denaturation at the upper solder layer with insufficient cross-linking at the solder-tissue interface. Such a process would also produce weak and unstable welds (Figure 6A). [50] Naturally, the intensity of heat generation is also defined by lasing parameters (i.e., irradiance, fluence, pulse duration, and irradiation mode).

Second, heat distribution throughout the solder is influenced by the solder’s thermal properties, namely the solder’s heat conductivity and diffusivity. In that respect, it has been reported that solders with an albumin content of ≥ 50% distribute heat more efficiently than solders with an albumin concentration of < 50% [83,84]. The efficiency of heat distribution in the high-albumin content solders is reflected by their denaturation pattern. The solder transmits heat to the base of the solder, where it causes denaturation. This allows homogeneous heat distribution across the full thickness of the solder, coagulating the entire solder layer, and the transmission of heat to the solder-tissue interface, facilitating cross-linking of albumins and tissue collagens. When the solder albumin content is too low, the generated heat is not transmitted to the base of the solder and therefore starts to denature at the solder’s superficial layer. The increase in back scattering with solder denaturation impairs transmission of heat to the entire solder layer [83,84]. As a result, high-concentration albumin solders produced stronger welds than solders with low album concentration (Figure 6B) [56].

Finally, the amount of energy required to denature proteins in the solder and the vascular wall is governed by the heat capacity of the solder and tissue, respectively [52, 84]. The heat capacity of albumin solders varies between species [52]. In comparison to human, porcine, and canine albumin, bovine serum albumin (BSA) has the lowest and narrowest denaturation peak [52]. Recently we found that BSA starts to denature at 58 °C and peaking at 81 °C [86]. Human serum albumin (HSA), on the other hand, possesses the highest and widest denaturation range (59-94 °C) [52]. Due to the lowest heat capacity, BSA solder requires the least energy (i.e., heat) to coagulate. Accordingly, welding with BSA solder is associated with the lowest risk of thermal damage compared to solders composed of albumin from other mammalian sources, and is therefore the most preferred solder protein for sLAVW [52]. On the basis of laser-solder interactions, welding strength is determined by (1) lasing parameters, (2) chromophore concentration, (3) solder source, (4) solder concentration, and (5) solder state.

#### 3.2.3. Summary of experimental results

**Figure 7.**
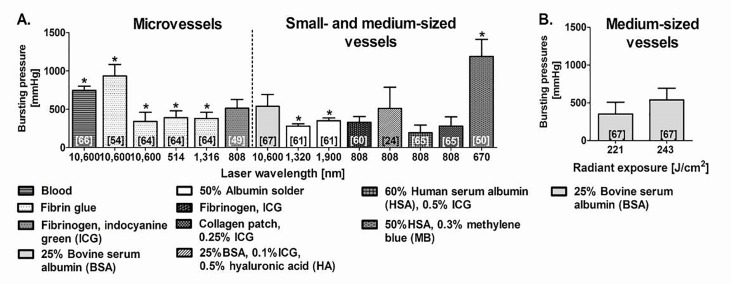
Summary of welding strengths achieved by sLAVR of micro-, small-, and medium-sized vessels performed at different laser wavelengths (A) and radiant exposures (B). In (B), sLAVR was performed in medium-sized vessels with a CO2 laser (λ = 10,600 nm) at an irradiance of 13.9 W/cm^2^. (*) indicates that stay sutures were used during sLAVR. Microvessels, small-, and medium-sized vessels correspond to vessel sizes of ≤ 1 mm (i.e., rat vessels), 1-2 mm (i.e., rabbit vessels), and 2-7 mm (i.e., pig vessels), respectively.

sLAVW increases welding strength by at least 25% compared to LAVW [40,41,61]. Figure 7A summarizes the welding strengths achieved with sLAVR using different wavelengths, different solder sources, and different solder compositions. In correspondence with the law of Laplace, sLAVR on small- and medium-sized vessels achieved lower bursting pressures than microvessel repairs. Despite the addition of solder, stay sutures were still required to support anastomoses in half of the studies (Figure 7A). Due to its low OPD, the CO_2_ laser was mostly used for microvessel sLAVWs, whereas the Nd:YAG (1,320 nm) and diode lasers (1,900, 808, and 670 nm) were commonly used for welding of small- and medium-sized vessels. The combined use of a diode laser, solder, and chromophore in sLAVW resulted in better patency, less thermal damage, and less thrombus formation compared to the Nd:YAG laser [62,67,87]. To optimize welding strength in CO_2_-mediated sLAVR of medium-sized vessels, Wolf-de Jonge et al. utilized a 2- or 3-pass scanning regime at increased radiant exposures (Figure 7B) [67].

As previously stated, the thermodynamic profile during welding chiefly determines welding strength. Of importance is the manner in which a thermal gradient evolves between the nucleation centers (there where light absorption occurs, i.e., the chromophore molecules) and the non-absorbing regions as well as the consequent pattern of heat diffusion. More homogenous heating of the solder/tissue at lower chromophore concentrations produces a narrower temperature gradient, which has been associated with stronger repairs than welds made at higher dye concentrations, where heating is more heterogeneous and characterized by steeper thermal gradients (Figure 6A, 8A) [50,56,63]. Alternative modulations of the thermodynamics during sLAVW include the separate application of chromophore and solder [57] and the employment of a solid solder film containing a chromophore gradient [58]. These modalities further increased welding strength [57,58]. The separate application of solder and chromophore, however, resulted in extensive thermal damage [57].

In addition to enhancing energy absorption, the color change of chromophores provides a visual cue to terminate irradiation or advancing lasing to an adjacent spot [50,63, 88,89]. ICG turns tan, brown, and black to indicate smooth drying, gritty drying, and carbonization, respectively [63,88, 89]. MB, on the other hand, turns white upon heating (i.e., the leucoform of MB), which additionally switches off MB- mediated heat production [50]. Hence, based on the leucoform transition, MB has an advantage over ICG.

**Figure 8.**
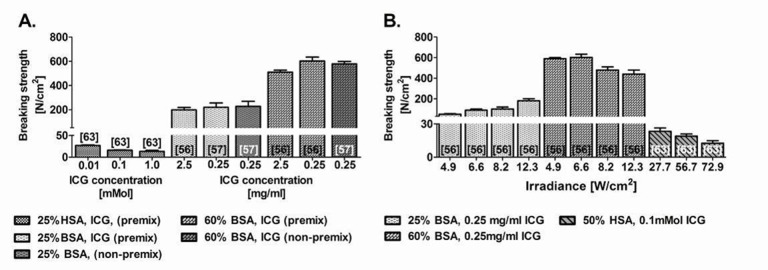
Summary of welding strengths achieved by sLAVR in medium-sized vessels (Ø = 2-7 mm, i.e., vessels of a pig) performed at different ICG concentrations (A) and a different irradiance (B). In (A) ssLAVR was performed with either bovine serum albumin (BSA) solder or human serum albumin (HSA) solder and irradiated with an 808-nm diode laser. In (B) ssLAVR was performed with 810-nm [63] and 808-nm [56] diode lasers. The numbers in brackets indicate the reference from which the data were obtained.

The variations of solders used in sLAVW are demonstrated in Figure 7A and 8. Blood and fibrinogen were the first solders used in microvascular sLAVW (Figure 7A) [54,64,66]. However, stay sutures were required to increase the welding strength. As previously stated (section 3.2.2), BSA has a lower heat capacity than HSA solder and thus requires less irradiance to optimize the welding strength (Figure 8A) [56,63]. Furthermore, on the basis of its superior heat conductivity and diffusivity, the 60% solid BSA film requires a lower irradiance and a shorter irradiation time than the 25% BSA solder (Figure 8B) [56]. Consequently, the solid solder exhibited a lower surface temperature (i.e., 85 °C) than the liquid solder (i.e., 125 °C) and induced less thermal damage [56]. The higher albumin concentration and the solid state of the solder support stronger cohesive bonding and better positioning on the bonding area, respectively, which further highlights the advantages of the 60% solid BSA solder. In comparison to 25% BSA sLAVR, the 60% solid solder film increased welding strength by 173% (Figure 8B) and exhibited thermal damage limited to the adventitial layer [56]. Modification of the solid solder in the form of a BioWeld ring produced intact sutureless end-to-end anastomoses. However, the rigidity of the solder resulted in changes of vascular compliance [90].

#### 3.2.4. Drawbacks of sLAVW

Regardless of the improvement in soldering strength and the reduction in thermal damage, sLAVW has mainly been performed in the context of in vitro sLAVR [24,50-53,55-58, 61-63,65,67,84]. Several important issues apply to in vivo sLAVA, including: (1) insufficient welding strength in small-to-medium-sized vessel sLAVA (Figure 7A), (2) inconsistency of results with liquid solder sLAVA as a result of solder leakage, (3) the rigidity of solid solder films that hinders vascular application, and (4) the solubility of the solder in a physiological environment. Pre-denaturing the solder prior to application decreases solder solubility but also reduces the welding strength [58,91,92]. Drenching a biodegradable scaffold in a liquid solder is considered a better solution to minimize solder solubility and leakage and to increase soldering strength. Furthermore, the flexibility of the scaffold is more favorable for vascular applications than solid solder films [58].

### 3.3. Scaffold- and solder-enhanced laser-assisted vessel welding (ssLAVW)

#### 3.3.1. Principal mechanism

ssLAVW comprises the use of a polymeric scaffold to enhance cohesive bonding of the liquid solder during sLAVW [93-103,108]. A semi-porous scaffold composed of biocompatible polymeric material is drenched in chromophore-containing solder, placed over the coaptation, and irradiated. The scaffold provides an intertwining fiber network to fortify the thermally-coagulated solder. To date, only poly(lactic-co-glycolic acid) (PLGA) and poly(ε-caprolactone) (PCL) have been used as reinforcement materials in experimental ssLAVW.

The use of a polymeric scaffold increases welding strength by 2-fold [97,102]. Moreover, the possibility of producing tube scaffolds allows the future application for end-to-end or end-to-side anastomoses of medium-sized vessels [58,97,102]. In addition to vascular anastomosis, ssLAVW can also be used as an immediate extra-vascular sealant [99,104].

#### 3.3.2. Laser-scaffold interactions

Principally, the thermodynamics in ssLAVW are similar to sLAVW. Lasing parameters and the solder’s optical and thermal properties remain pivotal for heat generation and the dynamics of heat distribution within the solder/scaffold coagulum. However, the scaffold physical properties (i.e., scaffold thickness and the degree of porosity) affect the SI. Scattering increases with increasing scaffold thickness and fiber density [97]. Due to the higher SI, ssLAVW requires higher radiant exposure than sLAVW to obtain optimal soldering strength [58,102].

Based on the laser-(solder)-scaffold interactions, the welding strength of ssLAVW is governed by (1) lasing parameters, (2) chromophore concentration, (3) solder and scaffold application procedure, (4) solder characteristics, and, most importantly, (5) the physicochemical characteristics of the scaffold.

#### 3.3.3. Summary of experimental results

**Figure 9.**
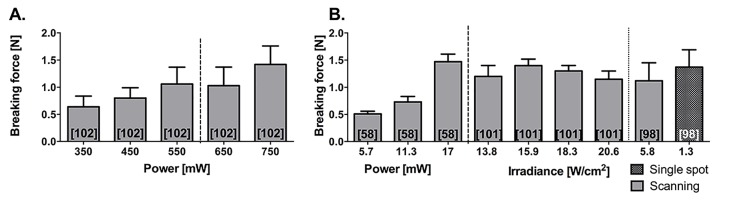
Breaking force obtained with ssLAVR performed at different powers (A) and irradiances (B) ssLAVR in [58,101,102] was performed with poly(lactic-co-glycolic acid) (PLGA) scaffolds, indocyanine green-containing 50% bovine serum albumin (BSA) solder and an 806-nm diode laser. The ssLAVR in [98] was performed with a poly(ε-caprolactone) (PCL) scaffold, a solder containing 48% BSA, 0.5% methylene blue, and 3% hydroxypropylmethylcellulose and a 670-nm diode laser. All experiments were performed on porcine aortic strips. The numbers in brackets indicate the reference from which the data were obtained.

**Figure 10.**
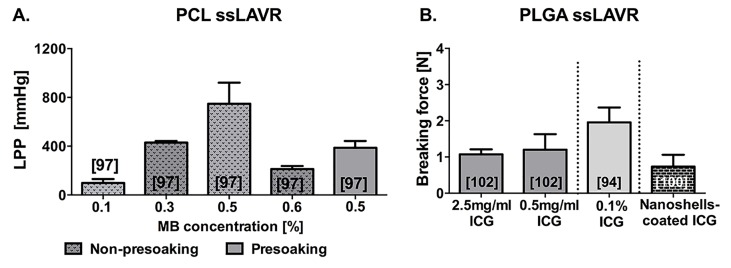
Summary of welding strength achieved with ssLAVR plotted as a function of methylene blue concentration (A) and indocyanine green concentration (B). In (A) ssLAVR was performed with a 50% bovine serum albumin (BSA) solder, poly(ε-caprolactone) (PCL) scaffolds, and a 670-nm diode laser. In (B) ssLAVR was performed with a 50% BSA solder, poly(lactic-co-glycolic acid) (PLGA) scaffolds, and an 806-nm diode laser [102]. SsLAVR in (A) was performed on porcine carotid arteries [97], whereas in (B) ssLAVR was performed on porcine [102] and rabbit aortic strips [94, 100]. The numbers in brackets indicate the reference from which the data were obtained.

Figure 9 and 10 depict the lasing parameters and chromophore concentrations as a function of welding strength, respectively. The majority of ssLAVW studies were performed in vitro as ssLAVR of small- and medium-sized vessels and used either a 670-nm or an 806-810-nm diode laser [86,93-103]. Figure 9 shows the improvement in welding strength with increasing power and irradiance. Due to the increased SI, ssLAVR required higher lasing parameters (Figure 8B vs. 9, respectively) and a higher chromophore concentration to achieve similar welding strength as sLAVR (Figure 6A, 8A vs. Figure 10, respectively). Consequently, ssLAVR exhibited more extensive thermal damage than sLAVR [58].

Next to lasing parameters and chromophore concentration, welding strength in ssLAVR is dictated by the application method of solder and scaffold (Figure 10A) [97,102]. Drenching the scaffold in a solder before applying it onto the coaptation reduces solder leakage [58,102]. However, scaffold impregnation likely causes heat generation at the anterior surface of the scaffold, which impairs heat distribution to the solder-tissue interface and therefore produces weaker welds than the non-presoaking technique [97,102]. In contrast to the scaffold presoaking technique, applying the scaffold to a solder-coated coaptation enables heat production at the solder-tissue interface and, thereby, more homogenous solder denaturation. Hence, this non-presoaking technique yielded stronger and more stable welds than the presoaking technique (Figure 10A) [97]. The non-presoaking technique, however, was associated with an increased risk of extensive thermal damage due to the distribution of heat to the vessel wall and the leakage of chromophore-containing solder through the coaptation [97,103]. The leakage and thermal damage issues can be circumvented by grafting chromophore-encapsulating nanoshells to the scaffold [100]. This recent technical advance in ssLAVR provides better control over heat deposition and eliminates the solder leakage-induced thermal damage.

**Figure 11.**
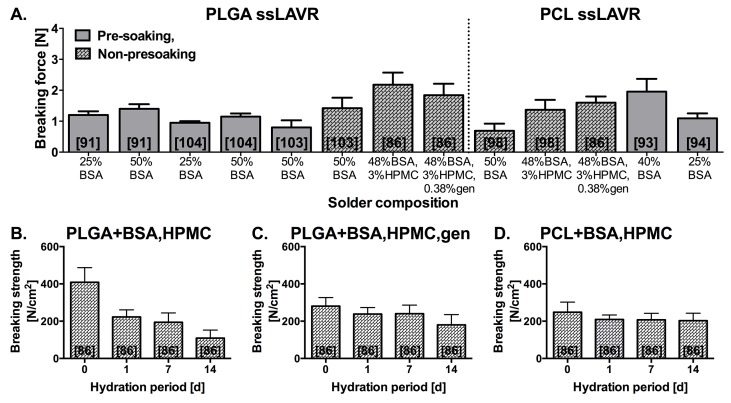
Summary of acute breaking force (A) obtained by poly(lactic-co-glycolic-acid) (PLGA) and poly(ε-caprolactone) (PCL) ssLAVR as a function of solder composition and solder application procedure. Post-hydration breaking strength of welds made with ssLAVR using PLGA (B,C) and PCL (D), plotted as a function of hydration time [86]. (*) represents the level of significance compared to the 0-d hydration subgroup, (†) indicates the level of significance versus aortas welded with PCL and bovine serum albumin (BSA)-hydroxypropylmethylcellullose (HPMC) solder at the respective time point, and (§) defines the level of significance between the respective subgroups in B (PLGA and solder-containing BSA-HPMC) vs. C (PLGA and solder-containing BSA-HPMC-genipin (gen)). In (A) ssLAVR was performed with 808-810-nm [91,93,94,103,104] and 670-nm [86,98] diode lasers. ssLAVR in B,C, and D were performed with a 670-nm diode laser. All experiments, except for [93,94], were performed on porcine aortic strips. In [93,94] ssLAVR was performed on rabbit carotid arteries. The numbers in brackets indicate the reference from which the data were obtained.

**Figure 12.**
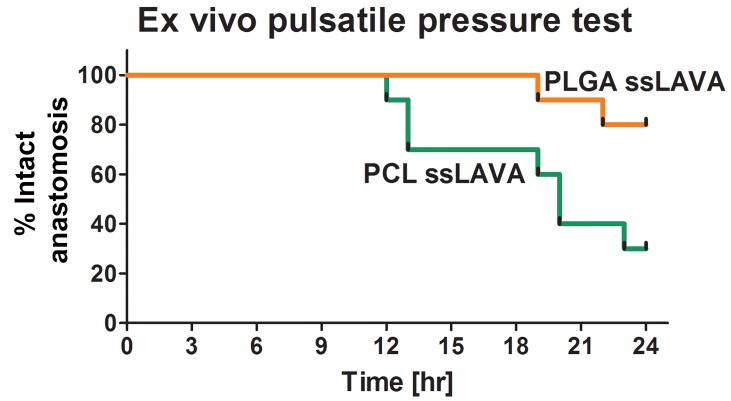
Kaplan-Meier-type plots of the percentage of intact arteries as a function of pulsatile perfusion time. Poly(ε-caprolactone) (PCL) ssLAVA was performed with the combination of an electrospun PCL scaffold and a solder containing 48% bovine serum albumin (BSA), 0.5% methylene blue (MB), and 3% hydroxypropylmethylcellulose (HPMC). Poly(lactic-co-glycolic-acid) (PLGA) ssLAVA encompassed the combination of an electrospun PLGA scaffold and a solder containing 48% BSA, 0.5% MB, 3% HPMC, and 0.38% genipin. ssLAVR was performed on porcine carotid arteries (external diameter of 3.9-5.3 mm) and completed with 11-spots irradiation with a 670-nm diode laser.

Figure 11 demonstrates the influence of solder composition and the scaffold’s thermo-mechanical properties on acute and post-hydration welding strength. As with sLAVR, a higher solder protein concentration produced stronger repairs than lower protein concentrations [91,93,94,98,102,103,104]. The reduction in solder leakage was achieved by semi-solidification of the solder. The addition of e.g., a gel-forming agent such as hydroxypropylmethylcellulose (HPMC) or hyaluronic acid (HA) increases solder viscosity and was associated with a 1.8-fold increase in welding strength [91,98]. Our recent results revealed that the highest acute welding strength was produced with the combination of semi-solid solder and the high melting point PLGA scaffold (148 °C) (Figure 11A). However, the hydrophillicity of the PLGA scaffold caused deterioration of welding strength when incubated in physiological buffer (Figure 11B). Scanning electron microscopy (SEM) analysis revealed a water-induced loosening of cohesive and adhesive bonds in post-hydration PLGA ssLAVRed aortas. The stability of PLGA ssLAVR was improved with the addition of the protein cross-linking agent genipin [107], which enhances cohesive and adhesive bonding and thereby increases the stability of the weld (Figure 11C). With the addition of genipin, 80% of the end-to-end sutureless PLGA ssLAVAed carotid arteries withstood a 24-h ex vivo hemodynamic test without bursting or leaking (Figure 12) [108].

In contrast to PLGA ssLAVR, aortas welded with a low melting point PCL scaffold (T_m_ = 60 ºC) were stable for up to 14 d of hydration in physiological buffer (Figure 11D) [86]. The stability of PCL scaffold under quasi-physiological conditions possibly resulted from the hydrophobicity of the scaffold material and the enforcing effect of the melted fibers on adhesive bonding [86,94]. Unfortunately, when a substantial amount of heat was produced, e.g., during 11-spot ssLAVA of end- to-end carotid artery anastomoses, the PCL scaffolds failed to sustain welding strength. The lasing modality apparently melted and weakened the PCL scaffolds, as a result of which only 30% of the anastomoses passed the 24-h pulsatile pressure test (Figure 12) [108].

**Figure 13.**
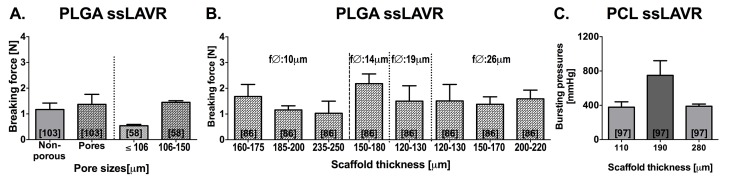
Summary of welding strengths achieved with poly(lactic-co-glycolic-acid) (PLGA) ssLAVR (A, B) and poly(ε-caprolactone) (PCL) ssLAVR (C) of porcine aortic strips. ssLAVR in (A) was performed with solvent-casted particulate-leached PLGA scaffolds, a solder containing 50% bovine serum albumin (BSA) and 0.5 mg/mL indocyanine green (ICG), and an 806-nm diode laser. ssLAVR in (B) was performed using electrospun PLGA scaffolds, a solder containing 48% BSA, 0.5% methylene blue (MB), and 3% hydroxypropylmethylcellulose (HPMC), and a 670-nm diode laser. ssLAVR in (C) was performed with electrospun PCL scaffolds, a solder containing 50% BSA and 0.5% MB, and a 670-nm diode laser.

Finally, welding strength depends on the physical properties of the scaffold, namely the degree of porosity, fiber diameter, and scaffold thickness (Figure 13) [58,86,97,103]. Using the same type of polymer and at comparable lasing- and solder properties, a highly porous scaffold decreases the SI and increases solder penetration, thereby yielding higher soldering strength than non-porous and tightly packed scaffolds [58,97, 103].

#### 3.3.4. Drawbacks of ssLAVW

**Figure 14.**
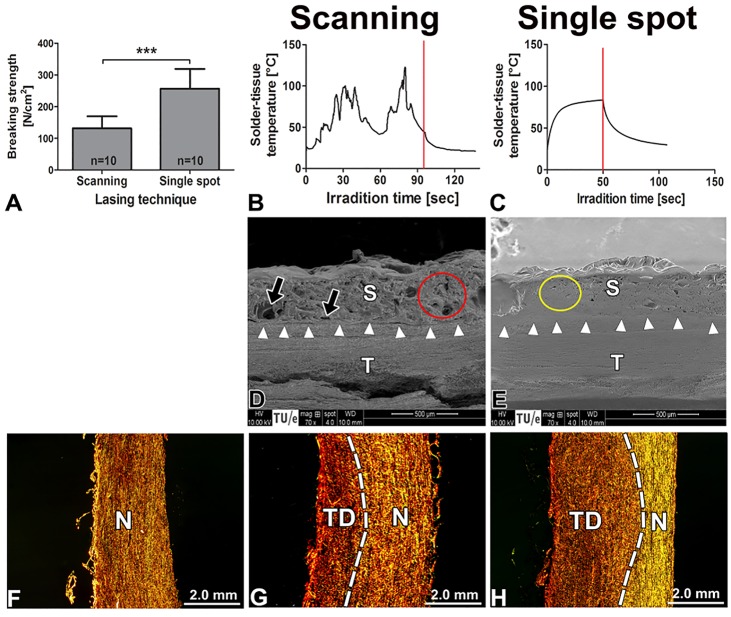
Welding strength (A), solder-tissue temperature (B, C), SEM images of the solder (S)–tissue (T) interface (D, E), and the degree of thermal damage (G, H) of scanning vs. single spot lasing modalities. (A) Presents the acute breaking strength of scanning vs. single spot lasing (n = 10/group). In B and C, the red line indicates the termination of laser irradiation. In D and E, white arrows are pointing to the solder-tissue interface, whereas black arrows indicate the gap at the solder-tissue interface. The red circle designates the fibrous solder/scaffold coagulum, while the yellow circle defines the compacted solder/scaffold coagulum. In (F-H), thermal affliction of vascular tissue (picrosirius red (PR)staining and polarization microscopy) is presented. Native (non-irradiated) aorta (F) was used as control. Low magnification images of PR-stained samples viewed under polarized light illustrate the extent of thermal damage (TD) between scanning (G) and single spot lasing (H).

As a consequence of scaffold-improved cohesive bonding, the adhesive bonding strength has become the weak point in ssLAVW [96,103]. The weak adhesive bonding is detrimental to the stability and patency of the coaptation [86,95,97,102]. Technical maneuvers to improve adhesive bonding (e.g., applying the scaffold to the solder-coated coaptation [97,103] or altering the lasing modality to single-spot irradiation [98]) resulted in substantial thermal damage (Figure 14)., Evidently, the challenge in ssLAVW lies in optimizing the balance between cohesive and adhesive bonding while minimizing thermal damage. ssLAVW is currently the most promising modality for clinical application and thus warrants further optimization.

### 3.4. Status quo of ssLAVW and possible solutions

#### 3.4.1. Improvements in cohesive bonding

The quality of cohesive bonding in ssLAVW is dictated by (1) the thermodynamics in the solder, (2) the composition of the solder, and (3) the thermo-mechano-physical properties of the scaffold.

Uniform solder denaturation is imperative for strong cohesive bonding. To enable uniform solder denaturation, heat evolution during welding should ideally start at the base of the solder. This type of heat evolution distributes heat throughout the entire solder layer and homogenously coagulates the solder, thereby producing strong cohesive bonds. Alternative modulations of the welding protocol aimed at achieving homogenous solder denaturation include scatter reduction [58,97,103], adjusting chromophore concentration and lasing parameters to enable homogenous heat distribution across the solder layer, [97,103] and the simultaneous delivery of radiant energy to the entire coaptation by e.g., intralumenal irradiation with circular diffusers [59]. Using a porous scaffold reduces scattering and thus enhances heat transmission across the solder/scaffold layer [58,97,103]. The chromophore concentration and lasing parameters dictate the deposition and distribution of heat across the solder layer. These parameters often have to be determined empirically. Next, uniform solder denaturation can be produced by simultaneous and homogenous irradiation of the entire coaptation. The homogenous delivery of radiant energy, however, increases the risk of extensive thermal damage and therefore has to be paired with the optimal combination of irradiances, irradiation time, and lasing mode.

For an ssLAVR model, single beam-emitting low-irradiance intermittent lasing is sufficient to provide homogenous heat distribution while limiting thermal damage [98]. However, for an end-to-end model, uniform radiant energy has to be obtained by 360° simultaneous irradiation [59]. In sLAVA, Ott et al. combined an intraluminal laser probe centered in a balloon catheter with the external application of a solder [59]. This modality homogeneously generates heat from inside the vessel wall, during which heat diffuses from the solder-tissue interface to the outer layer of the solder [59]. However, the internal irradiation is likely limited to small-sized vessels with a wall thickness of ≤ 0.3 mm [59,93,94,100]. In contrast to intraluminal irradiation, we believe that 360° external irradiation with e.g., several aiming beams attached to a clip probe could deliver simultaneous and uniform radiant energy and would be more applicable to all vessel sizes. This modality, however, may be associated with more extensive collateral thermal damage compared to intraluminal irradiation.

To limit thermal damage, external irradiation should be performed with a continuous-wave laser with cooling intervals (i.e., intermittent irradiation) [86] or with ultrafast irradiation with a pulsed laser (e.g., picosecond laser pulses) [109]. The combination of irradiation- and cooling intervals facilitates heat distribution to the solder-tissue interface and prevents excessive heat buildup in the underlying tissue. Recently, we have successfully reduced intermittent lasing–induced thermal damage without affecting the welding strength [86]. In contrast to continuous-wave lasers that distribute heat to the deeper layer via conduction, the pulsed laser enables sufficient heat generation at the solder-tissue interface without over-coagulating the solder superficial layer. The pulsed laser produced stable welds with minimal thermal damage [109].

Besides welding thermodynamics, cohesive bonding also depends on solder composition. Due to the thermal properties and higher density of proteins, which transmit heat more efficiently to the base of the solder, high protein concentration semi-solid solder is more beneficial for cohesive bonding than the lower protein concentration liquid solder.

Recently, we have shown that the cohesive bonding is governed by the physico-mechanical and thermal properties of the scaffold [86,97,108]. Porous scaffolds enhance the penetration of the solder into the scaffold and, thereby, improve cohesive bonding [58,97,102,103]. Moreover, cohesive strength is also influenced by the mechanical strength of the scaffold. The mechanical strength is dictated by the thickness of the scaffold and, in case of electrospun scaffolds, by the diameter of the scaffold fibers [86,97]. Hence, the ideal scaffold must have an optimal balance between fiber diameter, porosity, and scaffold thickness [86,97]. Furthermore, cohesive bonding is defined by the thermal properties of the scaffold. Ultrastructural analysis and mechanical tests on PCL and PLGA scaffolds confirmed that the high melting point of PLGA (148 °C) caused PLGA scaffolds to maintain fiber integrity after welding, yielding stronger welds than PCL scaffolds, which have a lower melting point (60°C) [86]. These results were obtained with irradiated free, solder-impregnated scaffolds and when the scaffolds were imposed on a vascular segment during ssLAVW. Nevertheless, welds made with PLGA scaffold are less resistant to physiological conditions [86], tend to degrade faster than PCL welds, and in doing so yield acidic by-products [110] (section 3.3.3). The instability of PLGA scaffolds in physiological solution can be improved by adding genipin to the solder [86] or by substituting PLGA with the more stable poly(lactic-acid) (PLA) scaffold [110].

#### 3.4.2. Improvements in adhesive bonding

In both solid solder sLAVW and ssLAVW, adhesive bonding is the weakest link [58,81,95,102]. Adhesive bonding is dictated by the thermodynamics at the solder-tissue interface [56,58,98,103], the solder properties [98], the melting point of the scaffold [94], and the hydrophilicity of the scaffold material [86].

Adhesive bonding predominantly depends on the evolution of heat at the solder-tissue interface [56,58,98,103]. To optimally cross-link albumin and tissue collagens, the temperature at the solder-tissue interface has to reach 62-67 ºC [100]. However, due to the difficulty of controlling heat evolution during welding, focusing heat at the solder-tissue interface increases the risk of inflicting extensive collateral (thermal) damage. Adjusting the chromophore concentration is the first step towards proper heat deposition. Second, fine-tuning the lasing parameters (i.e., laser probe, lasing mode, irradiance, and irradiation time) provides control over heat deposition and hence the extent of thermal damage. The combination of external application of solder (and scaffold) and 360° intraluminal irradiation, operated in low-irradiance continuous-wave mode, enables the generated heat to radiate from the solder-tissue interface to the solder superficial (outer) layer. Accordingly, this modality was shown to improve adhesive bonding and minimize thermal damage [59]. The intralumenal irradiation, however, is limited to micro- and small-sized vessels (Ø ≤ 1 mm or a wall thickness of ≤ 300 µm). Anastomoses of medium-sized vessels might require 360° simultaneous external irradiation instead (section 3.4.1). To ensure optimal adhesive bonding while preventing thermal damage, the 360° lasing has to be performed with either a continuous-wave laser operated at intermittent irradiation or by employing a pulsed laser (section 3.4.1).

Adhesive bonding also depends on the solder composition and the properties of the used polymer. As previously discussed (sections 3.2.2 and 3.4.1), solders containing a high albumin concentration allow more efficient heat transmission than solders with a low albumin concentration. With an optimal combination of chromophore concentration and lasing parameters, denaturation that starts at the solder base, as observed with solders containing high albumin content, potentially produces stronger adhesive bonding than the superficial denaturation seen with low albumin content solders. Furthermore, adhesive bonding can be increased by the addition of a protein cross-linking agent such as genipin, which chemically cross-links albumin to tissue collagens [107,111].

Recently we demonstrated an improvement in welding stability of PLGA ssLAVWed aortas by adding genipin to the semi-solid solder [86]. However, when scaffolds such as PCL were used, the semi-solid albumin solder was sufficient to produce stable aortic repairs. The hydrophobicity and low melting point of PCL scaffolds produce welds that retain their strength during 14-d hydration in physiological buffer. The hydrophobicity of PCL scaffolds also protects the welds from water-mediated deterioration of welding strength [86]. Moreover, with the welding temperature rising to ~80 °C, PCL fibers melt and coalesce with solder and tissue collagens, thereby enhancing adhesive bonding [86,93]. Unfortunately, when considerable heat was generated, i.e., during the 11-spots irradiation of end-to-end ssLAVA, PCL ssLAVAed arteries lost their welding strength during the 24-h pulsatile test and yielded a lower success rate compared to PLGA ssLAVAed arteries [108].

## 4. Photochemical laser-assisted vessel bonding (PLAVB)

Considering the thermal damage in the photothermal-based tissue bonding, researchers recently evaluated the use of photochemical reactions to create vascular anastomoses [48].

### 4.1. PLAVB procedure

For PLAVB, a visible light laser (e.g., green light laser) is employed in combination with a photosensitizer (e.g., Rose Bengal (RB)) that acts as a photochemical catalyst to induce immediate bonds and a water-tight seal between coaptated tissues. Prior to laser irradiation, the photosensitizer is applied on the external surface and inner surface of the proximal and distal vessel stumps, respectively. The proximal segment is then inserted into the distal segment and tissue apposition is maintained with a balloon catheter. The anastomosis is closed by continuous irradiation with a visible light laser [48]. The modality has also been successfully used in skin and nerve bonding [112,113].

### 4.2. Mechanisms of tissue bonding by PLAVB

The precise bonding mechanism in PLAVB remains elusive, but most likely involves the cross-linking of collagen type I [114]. In photochemical reactions, photosensitizer molecules absorb the incident light and transfer an excited state electron or energy to molecular oxygen, generating superoxide anion or singlet oxygen, respectively. These reactive oxygen species oxidize essential cellular structures and induce irreversible collagen cross-linking [48,114]. The laser fluence rate and photosensitizer concentration are the most important factors in the photochemical reactions [48,76]. Unlike photothermal reactions during (ss)LAVW, which require tissue or solder temperatures of at least 62 °C, photochemical reactions during PLAVB only increase the temperature to ~30 °C (from room temperature) and hence do not result in thermal damage [48,76,114 ,115].

### 4.3. Summary of experimental results

PTB has just recently been introduced in microvascular surgery as a potential alternative to microvessel welding. The relatively low irradiance used did not induce notable hyperthermia and yielded supraphysiological bursting pressures of 1,100 ± 150 mmHg without inflicting collateral (thermal) damage. Similar to (ss)LAVW, PLAVB provided an immediate liquid-tight seal and achieved 100% patency in an in vivo setting [48].

In related applications such as photodynamic therapy of solid cancers, the absorption of green light by RB causes the production of singlet oxygen that leads to cell damage, culminating in tumor cell death [116]. In contrast, a recent report on porcine skin PLAVB revealed that the photochemical reaction does not induce cell damage in this setting, which may be attributable to the low irradiance and the low RB concentration [117].

## 5. Future perspectives

The ultimate goal of laser-induced tissue bonding is to produce strong and durable anastomoses with minimal thermal damage to allow vessel healing. LAVW has evolved from microvascular anastomoses, which require stay sutures to support anastomotic strength, to sutureless anastomoses of medium-sized vessels that can withstand physiological and supraphysiological pressure. The addition of a chromophore-containing solder improves welding strength by creating an effect similar to gluing. This modality also omits the necessity of stay sutures and limits collateral thermal damage. A more recent advance in LAVW is the use of polymeric scaffolds, which fortify the coagulated albumin and enhance cohesive bonding. The reinforcement of cohesive bonding consequently leaves adhesive bonding as the weakest point. Regardless of the strength of the cohesive bond, a poor adhesive bonding capacity leads to unstable welds (i.e., the welding strength deteriorates under physiological conditions). An improvement in adhesive bonding (by altering the lasing regimen) resulted in increased post-hydration welding strength but inflicted more extensive thermal damage (section 3.4.2). Thus, prior to making the transition to the clinic, the challenge in ssLAVW lies in establishing the optimal balance between cohesive and adhesive bonding while minimizing thermal damage and establishing in vivo proof-of-concept.

Numerous studies on LAVW, sLAVW, and ssLAVW have shown that the enhancement of cohesive and adhesive bonding can be actualized by (1) the optimization of thermodynamics in the solder and at the solder-tissue interface, (2) the application of a high concentration semi-solid protein solder, and (3) the use of a scaffold that enhances both cohesive and adhesive bonding.

Homogenous and efficient heat distribution is fundamental in producing strong and durable anastomoses. The first step towards efficient thermodynamics and uniform solder denaturation is concentrating the radiant energy at the solder-tissue interface. This can be established by combining 360° circumferential irradiation with the use of a chromophore-grafted scaffold and a laser in intermittent irradiation or ultrashort pulse mode.

The intraluminal irradiation is likely the most suitable modality for a minimally invasive approach. Moreover, the application of a balloon catheter is advantageous to support the unbonded vessel coaptation [59]. However, the modality is limited to microvascular and small-sized vessel anastomoses. Medium-sized vessel anastomoses will typically require lasers with greater OPD or higher irradiances and a longer exposure time. Compared to the internal irradiation, the circumferential external irradiation has a broader application range. The laser probe for external irradiation can be tailor-made based on the vessel size and the anastomosis model (e.g., end-to-end, end-to-side, or side-to-side). The other lasing parameters, i.e., irradiance, irradiation time, and irradiation mode, should be determined empirically. A low-irradiance continuous-wave laser operated in an intermittent regime is beneficial for uniform heat distribution and the limitation of thermal damage [86], whereas ultrashort irradiation with pulsed lasers provides better control of heat deposition and more efficient heat transfer [107].

The combination of high-concentration semi-solid albumin solder and genipin obtained the highest acute and post-hydration welding strength. Additionally, in an in vivo setting, genipin has the potential to increase resilience to cell-mediated and enzymatic degradation [111,118]. Future application in the clinical setting should consider employing a concentrated autologous plasma protein [119-121]. However, the higher denaturation temperature of HSA might require higher lasing parameters, which increases the risk of inflicting more extensive thermal damage [52]. Considering the relatively high denaturation temperature of HSA, chitosan-based adhesives deserve further investigation as an alternative to albumin-based solder. In laser soldering of small intestine, chitosan adhesive has been recently introduced as a substitute for albumin solder. Compared to albumin, chitosan can be welded at a temperature of ~32°C. Furthermore, chitosan has a lower risk of triggering an allergic reaction, is more malleable, and is more stable under physiological conditions than albumin solders [122].

The main goal of adding a polymer scaffold is to strengthen the cohesive bonding. However, the scaffold should also provide stable welds and should not affect vascular healing. Considering the positive effects of PLGA on cohesive bonding and of PCL on adhesive bonding, a dual-layer scaffold containing an inner layer of PCL and an outer layer of PLGA is potentially the most suitable scaffold for ssLAVW. The inner layer melts with the coagulated solder and tissue collagen, providing robust adhesive bonding, whereas the outer thermo-stable layer supports strong cohesive bonding. A dual layer scaffold can be produced by electrospinning [123].

The clinical implementation of scaffolds, however, may require utilization of natural protein-based products such as collagen and elastin [124]. Although considerably more expensive than synthetic polymers, the natural protein scaffolds would be more biocompatible and would not produce any acidic by-products as is the case with PLGA. The employment of a collagen scaffold may eliminate the requirement of a solder, whereas the elastin fibers could further fortify collagen denaturation.

Following the improvement in anastomotic strength, the second step towards the clinical use of LAVA is restricting the extent of thermal damage. Although many studies have reported a normal healing process following full-thickness thermal damage, thermal damage extending beyond the internal elastic lamina is associated with complications such as intimal hyperplasia, thrombosis, and aneurysm formation [13,15,21, 34,38,71,72]. Partial thermal damage, limited to the adventitia and upper medial layer, is therefore considered the therapeutic goal.

The combination of a chromophore-embedding scaffold, circumferential laser irradiation, and ultrafast irradiation with a pulsed laser might reduce thermal damage. However, an unequivocal reduction in thermal damage is established by photochemical-based vascular anastomoses. PLAVB produces water-tight anastomoses via photosensitizer-induced chemical reactions between tissue molecules, which do not induce any temperature increases and corollary thermal damage. More importantly, PLAVB has been shown to produce strong and durable welds [48].

The enhancement of anastomotic strength in LAVA increases the utility and implementability of the modality in minimally invasive anastomoses of small and medium-sized vessels. When thermal damage can be significantly reduced, the application of ssLAVA may be expanded to distal arterial bypass surgery in the lower extremities, coronary artery bypass grafting, and microvascular (replantation and free flap) surgery [40].

## 6. Conclusions

The improvements in anastomotic strength and the possibility to omit stay sutures has brought ssLAVW closer to clinical application. Fundamental requirements to ensure successful clinical transition of ssLAVW include: homogenous heat distribution, high protein concentration semi-solid solder with the addition of a protein cross-linking agent, and a scaffold that enhances both cohesive and adhesive bonding. A lot of focus should also be placed on reducing thermal damage without decreasing anastomosis strength and following up with translational in vivo proof-of-concept studies.
